# Persistent *Plasmodium falciparum* infections enhance transmission-reducing immunity development

**DOI:** 10.1038/s41598-021-00973-5

**Published:** 2021-11-01

**Authors:** Ruth Ayanful-Torgby, Esther Sarpong, Hamza B. Abagna, Dickson Donu, Evans Obboh, Benedicta A. Mensah, Joshua Adjah, Kim C. Williamson, Linda E. Amoah

**Affiliations:** 1 Noguchi Memorial Institute for Medical Research, University of Ghana, Accra, Ghana; 2 University of Cape Coast, Cape Coast, Ghana; 3 Uniformed Services University of the Health Sciences, Bethesda, MD USA

**Keywords:** Immunology, Molecular biology, Diseases, Medical research

## Abstract

Subclinical infections that serve as reservoir populations to drive transmission remain a hurdle to malaria control. Data on infection dynamics in a geographical area is required to strategically design and implement malaria interventions. In a longitudinal cohort, we monitored *Plasmodium falciparum* infection prevalence and persistence, and anti-parasite immunity to gametocyte and asexual antigens for 10 weeks. Of the 100 participants, only 11 were never infected, whilst 16 had persistent infections detected by reverse transcriptase-quantitative polymerase chain reaction (RT-qPCR), and one participant had microscopic parasites at all visits. Over 70% of the participants were infected three or more times, and submicroscopic gametocyte prevalence was high, ≥ 48% of the parasite carriers. Naturally induced responses against recombinant Pfs48/45.6C, Pfs230proC, and EBA175RIII–V antigens were not associated with either infection status or gametocyte carriage, but the antigen-specific IgG titers inversely correlated with parasite and gametocyte densities consistent with partial immunity. Longitudinal analysis of gametocyte diversity indicated at least four distinct clones circulated throughout the study period. The high prevalence of children infected with distinct gametocyte clones coupled with marked variation in infection status at the individual level suggests ongoing transmission and should be targeted in malaria control programs.

## Introduction

The scale-up of malaria interventions over the past decade has led to decreased rates of malaria morbidity and mortality, but these declines have slowed in the past 3 years^1^. This plateau in malaria prevalence is attributed to factors such as high subclinical infections that act as potential reservoirs for transmission in endemic populations^2,3^. Renewed and intensified approaches are needed to successfully eliminate the disease. Reports from health facilities shows that Ghana has a high microscopic *P. falciparum* infection prevalence in children under 5 years, which ranges from 4 to 33% depending on the location^4^. Areas of high parasite prevalence can serve as infection hotspots that maintain transmission in neighboring communities with lower prevalence. In high parasite prevalence settings (endemic populations) where only a fraction of the infected individuals are thought to be responsible for most of the *Plasmodium* transmission, monitoring the dynamics of *P. falciparum* infections can aid in the identification of the reservoir source^5^.

As subclinical infections are not treated in most hyper-endemic areas, these individuals will continue to produce gametocytes, the transmissible form of the parasite^2,6,7^. Mature, stage V gametocytes first appear in the peripheral blood 10 days after committed merozoites emerge from the liver^6,8^, and immature gametocytes continue to be produced during each erythrocytic asexual cycle. Continual gametocytogenesis provides a consistent supply of mature gametocytes capable of undergoing sexual recombination once taken up in a blood meal by a mosquito^9,10^. Therefore, multiclonal parasites in subclinical infections can increase population diversity. Asexual clones of *P. falciparum* can persist for weeks at the subpatent or subclinical levels to provide a continuous source of gametocytes^11,12^. Monitoring the clonal dynamics of gametocytes provides insight into the clones available for transmission between individuals via mosquitoes and their spread through the community.

Exposure to different stages of the *P. falciparum* parasite contributes to the acquisition of stage-related immunity. Antibodies elicited against the various stage-specific antigens serve as serological markers that can be used to measure exposure and test for anti-parasite or transmission-reducing activity^13,14^. The levels of host IgG and IgM antibodies against parasite antigens at the individual or population level provide an important metric to monitor infection progression and transmission dynamics. Antibody levels are also influenced by the age of the host and transmission intensity^15,16^. It has been shown that for an individual to be protected against clinical malaria, periodic infections are needed, even if they are sporadic or subclinical^17^. Protection against clinical malaria involves various mechanisms that contribute to parasite clearance. Several parasite antigens have been implicated in protection, including the conserved region III–V of PfEBA175 (EBA175RIII–V), which is expressed on the surface of the merozoite. Anti-EBA175RIII–V responses have been shown to prevent red blood cell (RBC) invasion^18,19^. While anti-gametocyte immunity can prevent the completion of the parasite development in the mosquito, thereby reducing or preventing malaria transmission^20^. Specific epitopes of Pfs48/45 and Pfs230 antigens, which are expressed during gametocyte development and exposed on the extracellular surface of the gamete during the transition to zygote formation have been shown to be the targets of transmission-blocking monoclonal antibodies^21^. Recently, tools have been developed to evaluate gametocyte production, maturation, and immunity within the human host and the contribution of this immunity in reducing transmission needs to be tested in the field^22–25^.

The effect of subclinical *P. falciparum* infections on malaria transmission is difficult to evaluate as neither these infections nor mosquito bite exposure are routinely quantified in endemic populations. Closely monitoring parasite infection dynamics including identifying and targeting carriers with persistent infections could break the transmission cycle in the defined population. This study assessed *P. falciparum* infection dynamics at the individual level and monitored infection heterogeneity and sexual-stage clones during the off-peak season. Additionally, the association of infection frequencies and antibody responses to recombinant EBA175RIII–V asexual stage and gametocyte-specific Pfs48/45.6C and Pfs230proC were examined.

## Results

This study evaluated *P. falciparum* infection dynamics in 100 children aged 6–12 years just after the peak malaria season in Ghana. Samples were collected every 14 days for 10 weeks; children present on five or more sampling days were included in the analysis. Feverish children with high body temperature were referred for clinical management. Monthly hemoglobin levels were assessed for each participant to monitor anemia prevalence in the study population. *Plasmodium falciparum* infections were detected at both patent and subpatent levels. Parasite positivity was also assessed using RDT kits (Table 1). Additionally, host antibody titers against the sexual stage, Pfs48/45C6 and Pfs230proC; and asexual, EBA175RIII–V stage, recombinant parasite antigens were evaluated monthly and compared with the parasite and gametocyte carriage (Figs. 3, 4, 5, 6).

**Table 1:** Clinical characteristics of the population and infection dynamics during the sampling time points.

Parameter	Day 0		Day 14		Day 28		Day 42		Day 56		Day 70		p value
	n		n		n		n		n		n		
Sex (female/total)		59/100		57/98		58/99		57/100		56/98		47/80	
**Axillary temperature (°C)**	100		91		92		98		96		80		
Mean (SD)		36.5		36.3		36.4		36.5		36.4		36.4	0.2587^**ɞ**^
Range		(34.6–39.1)		(26.2–39.1)		(34.7–39.3)		(35.1–37.6)		(35.4–37.3)		(33.1–38.0)	
*HB (g/dl)	100				92				96		80		
Mean (SD)		11.6 (1.6)				11.5 (1.3)				11.6 (1.4)			0.7933^**ɞ**^
Range^∞^		(7.7–15.2)				(8.1–14.3)				(6.9–15.0)			
RDT	100	66/100									80	32/80	
**Parasite density**	100		91		92		98		96		80		
Positive		25		21		30		37		41		24	**0.0445**^**ɞ**^
Parasite density GM (CI)/µl		682 (382, 1219)		1395 (829, 2320)		1532 (887, 2644)		597(378, 943)		765 (529, 1106)		815 (417, 1591)	
Highest parasite density/µl		9000		8440		17,960		12,640		16,760		35,720	
Parasite density log10 GM, CI		2.8 (2.6, 3.1)		3.1 (2.9, 3.3)		3.1 (2.9, 3.4)		2.7 (2.5, 2.9)		2.8 (2.7, 3.0)		2.8 (2.6, 3.1)	
**Microscopic**	94		91		92		98		96		80		
Prevalence (%), CI		27 (25, 44)		23 (21, 40)		33 (30, 50)		38 (37, 57)		43 (36, 56)		30 (29, 51)	**0.0461**^**ɤ**^
Persistent (n)		1											
**Molecular (RT-qPCR)**	94		91		92		98		96		80		
Positive		57		63		65		64		55		46	
Prevalence (%), CI		61 (51, 71)		69 (58, 78)		71 (51, 71)		65 (55, 74)		57 (47, 67)		58 (49,71)	0.2560^**ɤ**^
Persistent (n)		16											
**Subpatent infections**^#^		32		42		35		27		14		22	
Prevalence (%)		34		46		38		28		15		28	** < 0.001**^**ɤ**^
**Gametocyte (IP) (RT-qPCR)**^**~**^	58		63		60		64		55		49		
Positive		38		30		33		33		43		26	
IP prevalence (%), CI		67 (58, 82)		48 (34, 60)		52 (40, 66)		52 (38, 64)		78 (69, 90)		57 (45, 73)	**0.0073**^**ɤ**^
Persistent (n)		2											
Clones (n)		4		5		5		7		10		8	
MOI		1		1.3		1.4		1.2		1.6		1.5	0.4159^**ɞ**^
**No infection**													
Uninfected (n)		11											

*n* (number of samples), participants absent at a time point were not included. *Two participants have variant hemoglobin genotypes (SS and SC genotypes).

^∞^Minimum recorded parasite density was set to 80 µl per whole blood. ^~^Proportion of *P. falciparum* infections with gametocytes detected by molecular method.

^#^Proportion of individuals with only submicroscopic parasites (the difference between the number of molecular and microscopic infections).

*IP* infected population (only samples positive for 18S rRNA were evaluated for gametocytes).

p values among time points were tested using one-way ^ɞ^ANOVA and ^ɤ^Chi-Squared. p values < 0.05 were considered significant as indicated in bold.

### Clinical and infection parameters

The number of female (59%, n = 58) participants in the study was slightly more than the males. The monthly mean hemoglobin (Hb) level was estimated at 11.4 g/dl, and one-third of the participants (31/99) had low Hb levels (< 10 g/dl). Nonetheless, the Hb levels did not differ between time points (0.7933) or infection groups (p = 0.2296) (Table 1; Supplementary Table 1). Two participants, one with Sickle-cell disease (HbSS) and one with sickle cell as well as variant hemoglobin C (HbSC), had persistently low hemoglobin levels (< 9.5 g/dl) and were referred for clinical management. The mean axillary temperature of the participants on each sampling day was normal (< 37 °C). Three participants with high body temperatures (38.3–39 °C) were referred for clinical management. The PfHRP2 RDT positivity rate in the study population was 66% at the baseline (day 0) and 40% on the last sampling day (day 70). Participants that tested positive for malaria by HRP2 RDT but had no fever were not referred for clinical management. The geometric mean parasite densities in the population ranged from 597/µl to 1395/µl of whole blood over the six-time points (Table 1). A participant was excluded from the study after D0 due to malaria unrelated illness (Supplementary Table 2).

### Infection dynamics

**Figure 1:**
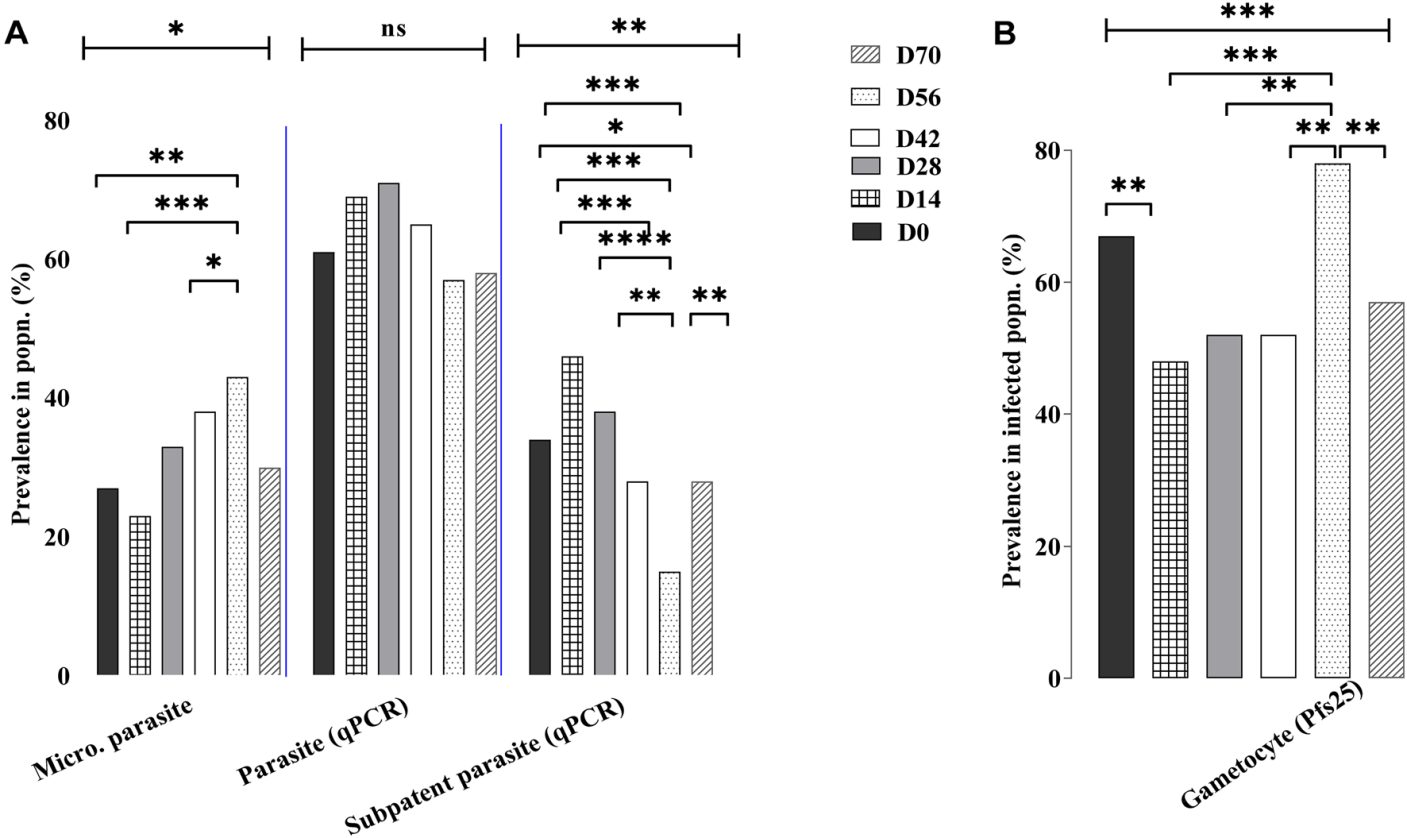
*Plasmodium falciparum* infection dynamics in the population. (**A**) The prevalence of parasites detected in the study population at each time point [Day (D)] using microscopy (Micro) and *Pf 18srRNA* reverse transcriptase-quantitative polymerase chain reaction [RT-qPCR (qPCR)]. Infections detected only by *Pf18S rRNA* using RT-qPCR, not microscopy were considered subpatent. Microscopic (Micro) parasite prevalence differed slightly (p = 0046) between sampling days. The prevalence of individuals infected only at the submicroscopic (Subpatent) level differed significantly (***p < 0.001) between time points. (**B**) Gametocyte prevalence detected in the infected population using *Pfs25* RT-qPCR also differed significantly (***p = 0.007). Analysis for all time points was by Chi-square and between time points by two sample tests of proportions (^ns^p > 0.05, *p ≤ 0.05, **p ≤ 0.01, ***p ≤ 0.001).

Parasite infections detected by *Pf18s rRNA* transcript levels were high and only 11 children remained aparasitemic through the entire study period. At each sampling time point over half of the participants (57–71%) were infected (Fig. 1) and 23–43% of the population had parasites detectable by microscopy. However, only one participant had parasites detectable by microscopy at all time points demonstrating fluctuation in parasite levels in individuals, even though at the population level parasite prevalence and microscopic parasite density remained relatively steady (Fig. 1A; Table 1). Although there was no significant change in total parasite prevalence, the microscopic prevalence was significantly higher on D56 (p ≤ 0.05) and there was a corresponding decrease in submicroscopic prevalence. This indicates that at this time point, more of the infected children had circulating parasitemia that was above the cut-off for microscopic detection (~ 80 parasites/µl).

### Gametocyte infection dynamics

Gametocyte prevalence in the infected population was also high ranged from 48 to 78% (n = 350) on the sampling days. Gametocytes were detected by microscopy in three infections and two children had gametocytes that persisted over the duration of the study (Table 1). Whereas gametocytes were not detected at any time point assessed during the study in fifteen (17%) of the infected individuals. Not surprisingly, most of the children (12/15) that never had detectable gametocytes were also rarely infected. In these 15 children, asexual parasites were detected just one to three times during the 6 visits and were mainly subpatent (Supplementary Fig. 1). However, of the 22 children that only had subpatent infections, 45% had gametocytes detected in at least one visit which is similar to the gametocyte prevalence in the study population (58%), but slightly lower than the prevalence in patent infections (64%, p = 0.013). Gametocyte detection during clinical malaria has been associated with low Hb levels ^26^, but in these asymptomatic children, Hb levels did not differ significantly between those with or without gametocytes or among the time points (Table 1; Supplementary Table 1). We further compared gametocyte densities (extrapolated from *Pfs25* transcript levels) in all the patent (n = 178) and subpatent (n = 172) infections (Supplementary Fig. 2) and found the total mean densities were similar (p = 0.8551). Although, when comparing parasite and gametocyte densities in the same sample there was a significant correlation (r = 0.3691, p < 0.0001) (Supplementary Fig. 3). More importantly, the gametocyte densities in 20% (39/198) of all the samples tested were above the minimum limit needed for sexual reproduction of 2 gametocytes/μl blood, the approximate volume of a mosquito blood meal (Supplementary Fig. 2). In total, 23% (26/114) of the patent and 15% (13/84) of the subpatent infections had > 2 gametocytes/µl blood, indicating that microscopy cannot be used promptly to identify potentially infectious individuals.

### Gametocyte diversity

To evaluate gametocyte diversity, gametocyte clones were identified based on RT-PCR amplicon size of polymorphic region 3 of gametocyte-specific gene *Pfg377*^27^. Eleven distinct clones (180, 200, 240, 260, 280, 300, 320, 340, 360, 380 and 400 bp) were observed during the study (Fig. 3; Supplementary Table 3). A quarter (25%, n = 149) of the gametocyte infections assayed were polyclonal and two individuals harbored three concurrent distinct clones at one time point. Overall, the observed gametocyte MOI was low (1.0 to 1.7) in the gametocyte carriers tested (Table 1), with no significant difference in MOI identified between any two time points (One-way ANOVA, p = 0.4159). Four clones (320, 340, 360, and 380 bp) dominated the infections when assessed by occurrence and were observed throughout the study. The first three time-points (day 0 to 28) were less diverse in terms of clonal distribution with the detection of only 4–5 clones per timepoint. Moving further into the off-peak season (day 42 to 70) clones < 300 bp began to be detected. Interestingly, all the infections with the < 300 bp amplicons appeared in polyclonal infections consistent with the introduction of new parasite strains into the population. The day with the highest number of distinct clones (D56, n = 10) also had the highest prevalence of both microscopically detectable infections and submicroscopic gametocytes.

The persistence of clones in individuals through the study period was also assessed. Most of the infected individuals assayed with circulating gametocytes at two or more time points had at least one distinct clone at each time point (21/24) (Supplementary Table 3). Only one individual had just a single clone detected through the course of the study and six individuals had the same clone detected in two consecutive samples. These findings demonstrate marked variation in parasite dynamics within an individual over the course of 10 weeks that would not be captured in cross-sectional studies. Gametocyte clonal diversity from collection to collection suggests that there is ongoing transmission in the community and that individuals are being infected, then clearing that clone and being reinfected with another clone circulating in the population.

### Antibody dynamics

**Figure 2:**
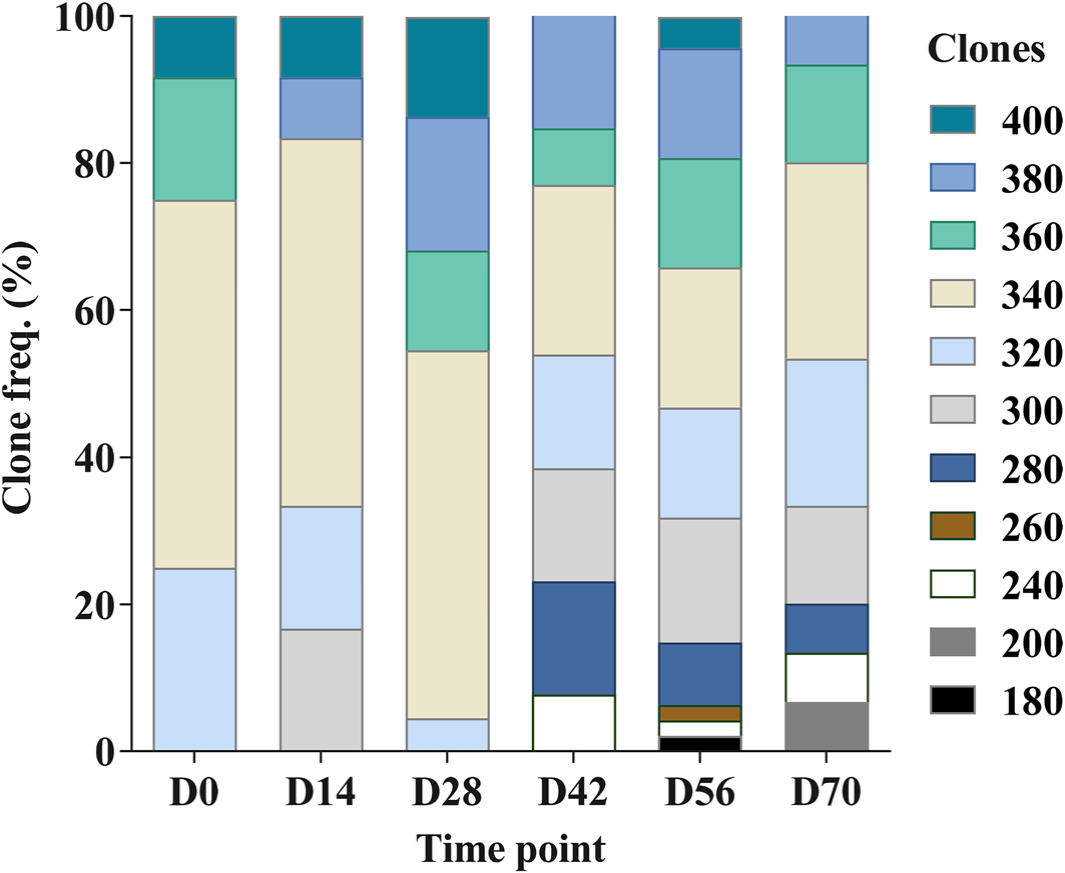
Distribution and duration of circulating gametocyte clones. At each time point analyzed [Day (D)] the different gametocyte clones (n = 11) detected in the subpopulation (n = 149) of infected samples tested are shown. Clonal frequency and distribution differed between time points. The highest number of diverse clones were observed on days 56 and 70. Clones below 300 bp (180–280) were least represented and observed only after day 28.

**Figure 3:**
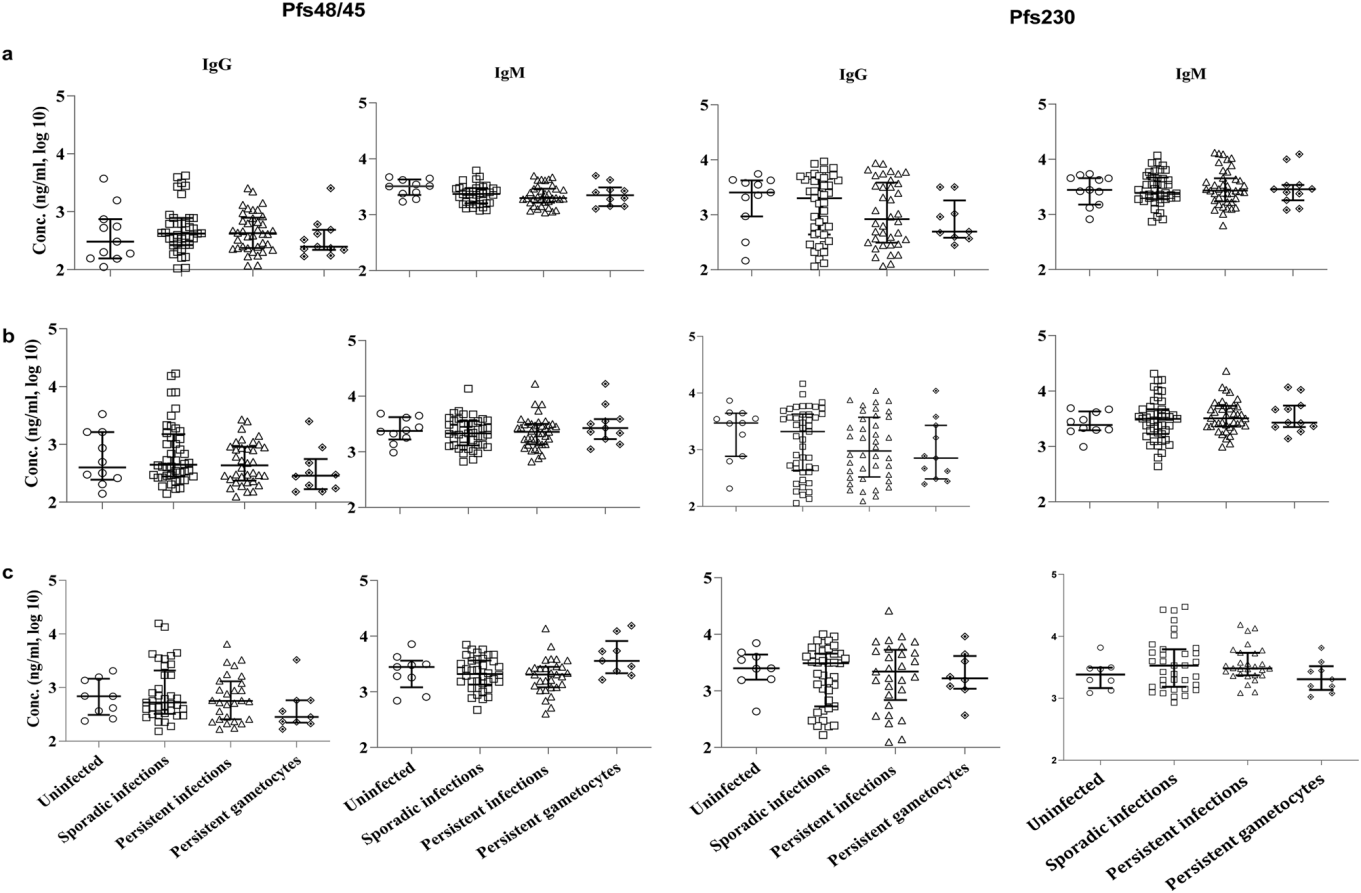
Antibodies against gametocyte antigens Pfs48/45.6C and Pfs230proC over the course of the study. Infectivity status did not influence the concentrations of IgG and IgM antibodies against gametocyte antigens, Pfs48/45.6C and Pfs230.proC. IgG and IgM antibodies levels (ng/ml, log 10) specific for gametocyte antigens, Pfs48/45.6C and Pfs230.proC were measured monthly [days 14 (**a**), 42 (**b**), and 70 (**c**)] in plasma from all the participants. The antibody concentrations obtained at each time point are plotted in groups based on overall infectivity status, uninfected (n = 11), sporadically infected; ≤ 4 infections (n = 49), persistently infected; ≥ 5 infections (n = 39), and persistent gametocytes; ≥ 5 episodes (n = 11) of gametocytemia. The median and interquartile range of the antibody concentrations are shown and differences between groups were analyzed by one-way ANOVA. The p values (p = 0.0631–0.994) are listed in Supplementary Table 4.

**Figure 4:**
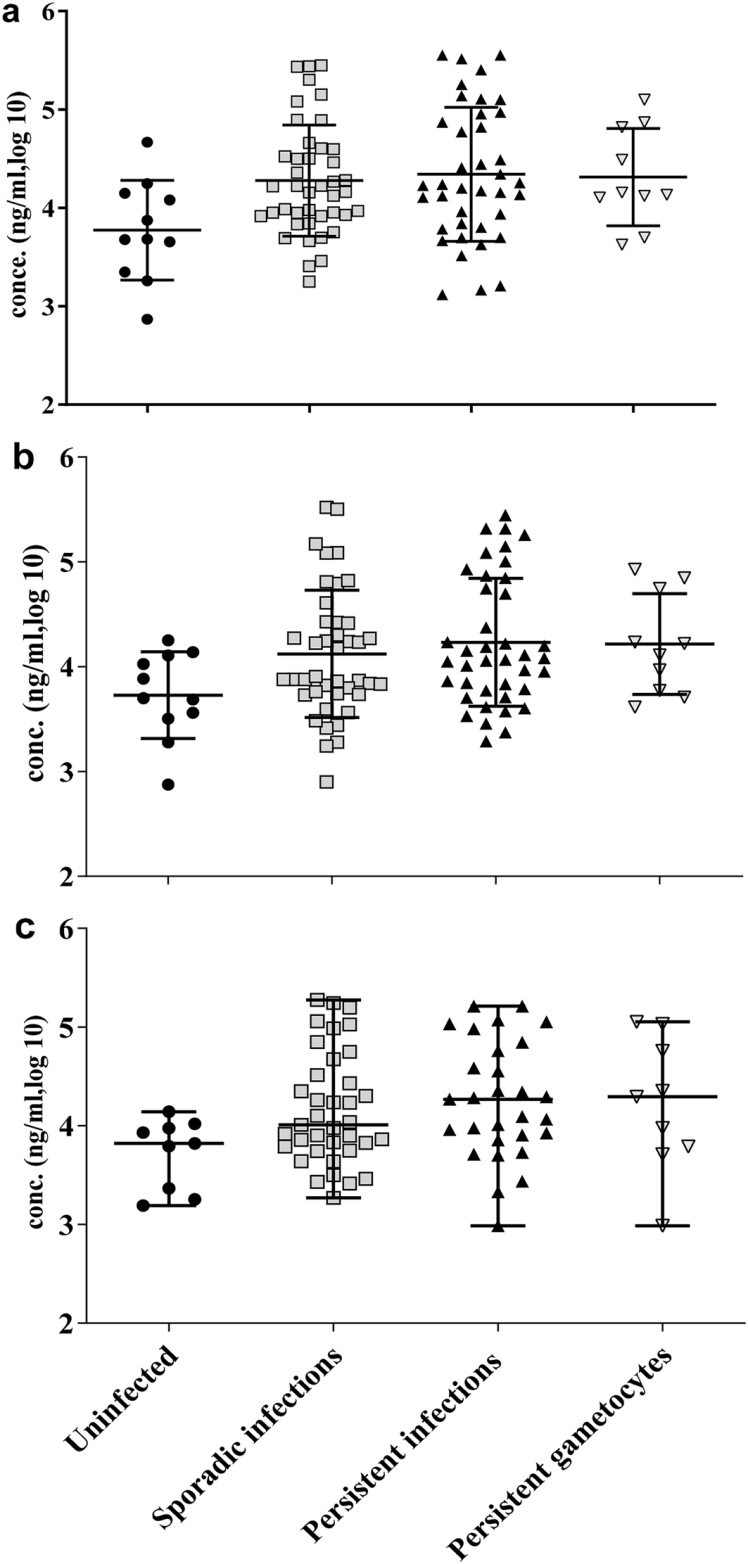
Antibodies against the asexual antigen EBA175 RIII–V over the course of the study. Anti-EBA175RIII–V IgG antibody concentrations (ng/ml, log 10) were measured monthly [days 14 (**a**), 42 (**b**), and 70 (**c**)] in plasma from all the participants. The antibody concentrations obtained at each time point are plotted in groups based on overall infectivity status. Uninfected (n = 11), sporadically infected, ≤ 4 infections (n = 49), persistently infected ≥ 5 infections (n = 39), and persistent gametocytes, ≥ 5 episodes (n = 11) were assessed using *P. falciparum Pf18S rRNA* and *Pfs25* transcripts for the detection of total parasite and gametocyte infections respectively. The median and interquartile range is shown and one-way ANOVA did not detect differences between the groups. The p values (p = 0.1647–0.7274) are listed in Supplementary Table 4.

**Figure 5:**
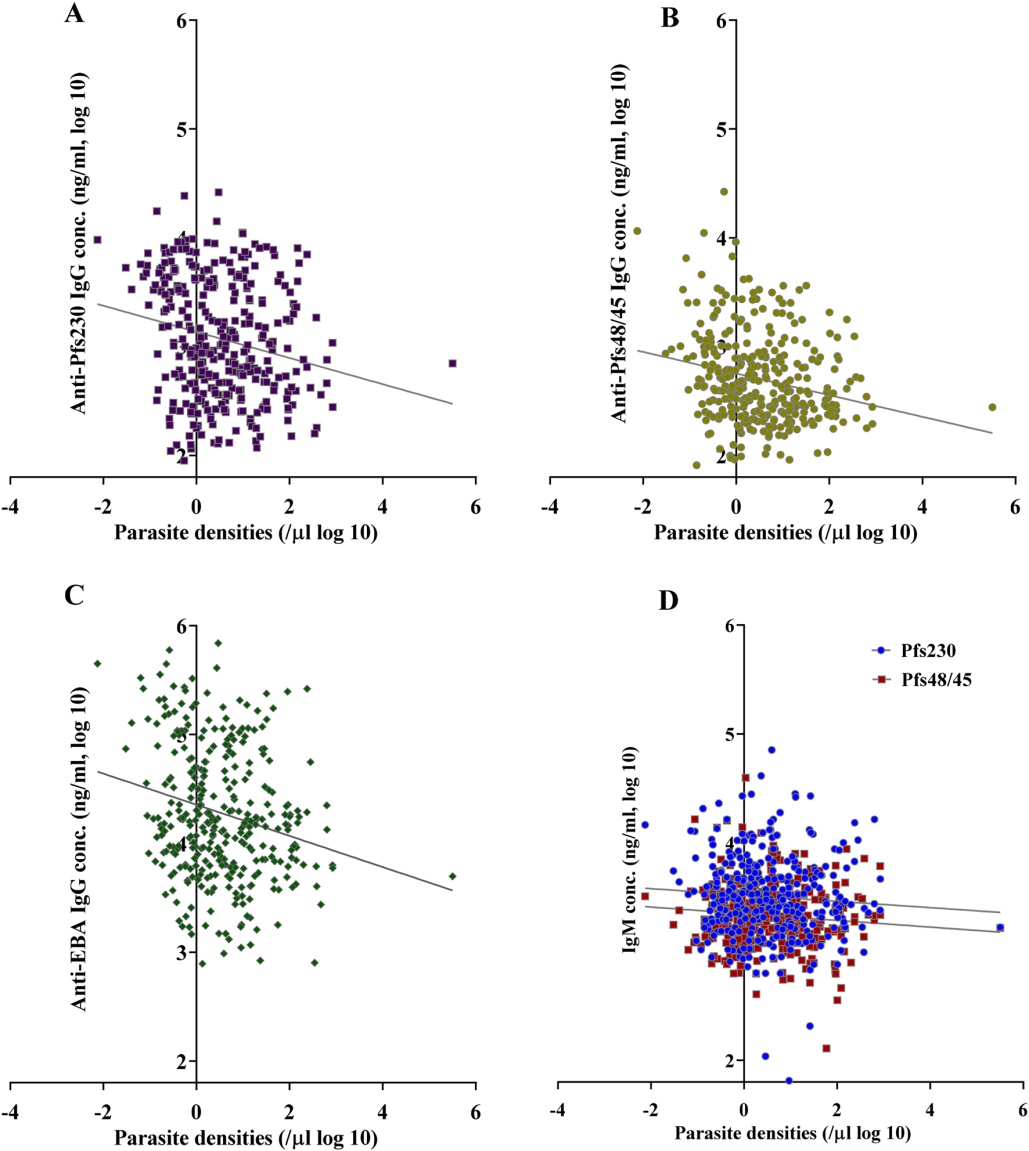
IgG antibody titers inversely correlate with parasite densities. Comparison of parasite densities estimated from *Pf18s rRNA* RT-qPCR analysis and the corresponding IgG (**A**–**C**) titers against Pfs230proC (**A**), Pfs48/45.6C (**B**) and EBA175RIII–V (**C**), and IgM (**D**) titers against Pfs230proC and Pfs48/45.6C. IgG, not IgM, responses were significantly inversely correlated with parasite densities. IgG Pearson r = − 0.2109**** (anti-Pfs230), r = − 0.2252**** (anti-Pfs48/45) r = − 0.2143**** (anti-EBA175). IgM Pearson r = − 0.07071 ns (Pfs230) and r = − 0.1010 ns (Pfs48/45). ^ns^p > 0.05, ****p ≤ 0.0001.

**Figure 6:**
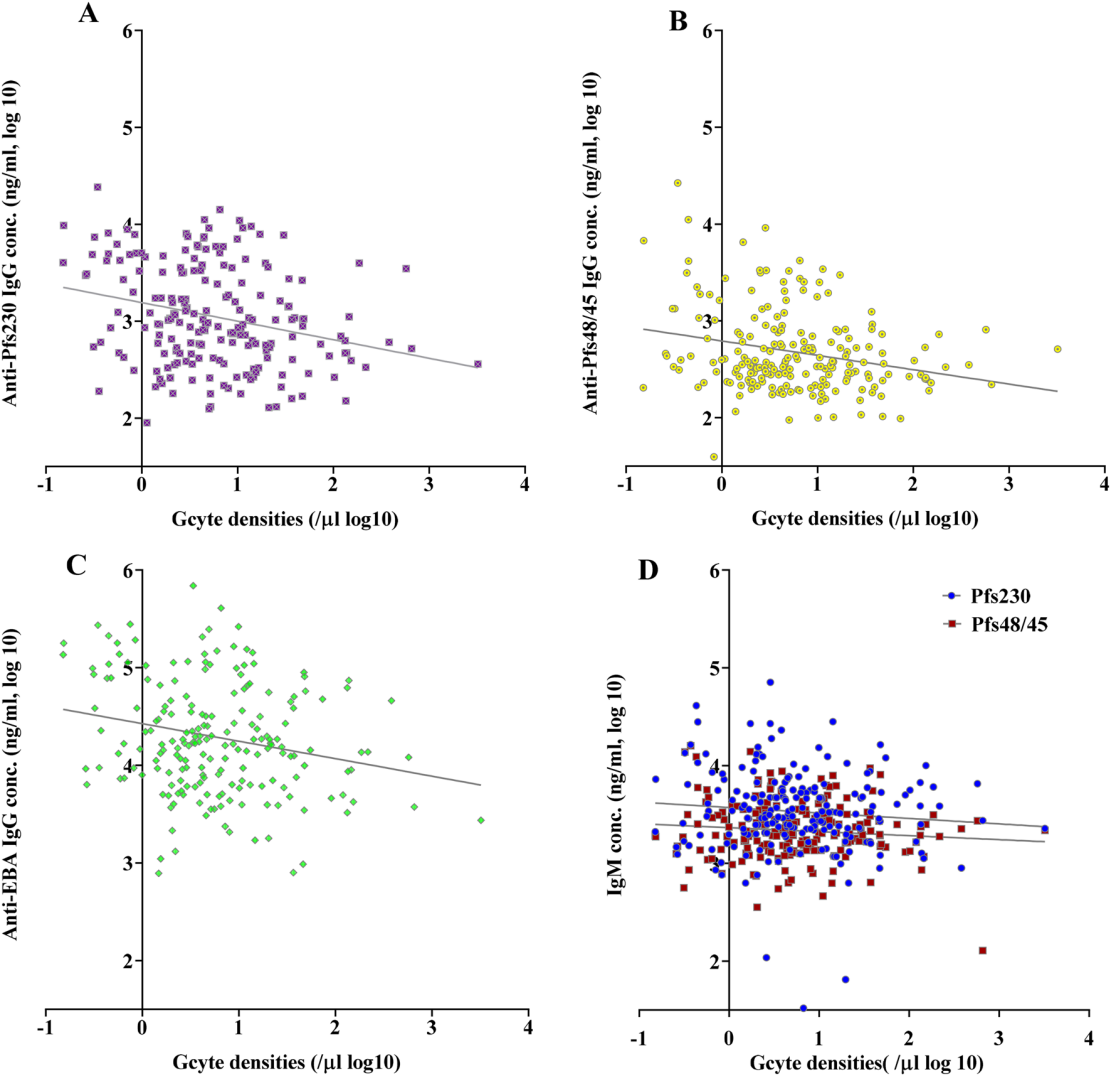
IgG antibody titers inversely correlate with gametocyte densities. Comparison of gametocyte densities estimated from *Pfs25* RT-qPCR analysis and the corresponding IgG (**A**–**C**) titers against Pfs230proC, Pfs48/45.6C and EBA175RIII–V, and IgM (**D**) titers against Pfs230proC and Pfs48/45.6C. IgG, not IgM, responses were significantly inversely correlated with gametocyte densities. IgG Pearson r = − 0.2656*** (Pfs230), r = − 0.2505*** (Pfs48/45) r = − 0.2144** (anti-EBA175). IgM Pearson r = − 0.0929 ns (Pfs230) and r = − 0.1033 ns (Pfs48/454). ^ns^p > 0.05, **p < 0.01, ***p ≤ 0.001.

To evaluate whether there was a correlation between infection status and anti-IgG and anti-IgM responses to gametocyte-specific antigens (Pfs48/45.6C and Pfs230proC) or anti-IgG to the asexual antigen (EBA175RIII–V), the participants were divided into four groups based on the parasite and gametocyte persistence. (1) Participants that remained uninfected throughout the study (n = 11). (2) Those with sporadic infections (≤ 4 episodes, n = 49). (3) Those with persistent infections (≥ 5 episodes, n = 39) or (4) a subpopulation of the persistently infected group that also had persistent gametocyte carriage were also analyzed separately (≥ 5 episodes, n = 11) (Figs. 2, 3; Supplementary Table 2). Surprisingly, no difference (p ≥ 0.0631) was observed in the measured Pfs48/45.6C or Pfs230proC antibody responses in the four groups at any time point (Fig. 3; Supplementary Table 4). Mean antibody titers were also not different between samples with and without detectable gametocytes (Supplementary Table 5). Similarly, no significant difference in mean IgG responses to the EBA175RIII–V antigen was observed (Fig. 4). However, anti-EBA175RIII–V titers were lower in the uninfected population consistent with the assumption that parasite exposure is needed to maintain high titers. Altogether, the data suggest that previous parasite exposure was sufficient to maintain antibody responses to limit parasite growth, but not confer sterilizing immunity to 89% of the study population. In a further comparison of antibodies titers and parasite or gametocyte density (Figs. 5, 6), there was a significant inverse correlation between parasite and gametocyte densities with IgG, not IgM, titers against all three antigens.

## Discussion

Both asexual and sexual stage parasite dynamics were tracked in asymptomatic school children using microscopy and molecular methods every 2 weeks through a 70-day study period, from November 2017 to January 2018, which is after peak malaria season in this region. Infection prevalence was high, with only a tenth of the participants remaining infection-free through the study period. However, there was marked individual variation in both asexual and sexual stage parasite carriage. This pattern of high asymptomatic parasite prevalence in the population and individual variation is common in endemic areas^28,29^. To better understand the underlying transmission dynamics, this work focused on the sexual stages of the parasite by tracking gametocyte carriage, diversity, and immunity.

Waxing and waning parasitemia and gametocytemia in a majority of the study participants is consistent with partial immunity coupled with ongoing transmission^30^. Ongoing transmission is also supported by the pattern of gametocyte clonal diversity observed. Eleven distinct gametocyte clones circulated in the population over the course of the study, but the MOI was low (< 1.5) and the same gametocyte clone was rarely detected in consecutive samples. These findings suggest that following infection with a particular clone, parasites emerge from the liver, invade RBCs and continue asexual replication while producing a subpopulation of gametocyte committed parasites each 48-h cycle. The erythrocytic cycle continues until the infection is controlled and that clone is cleared by the immune response. When the person is infected with another clone the cycle of increasing parasitemia and gametocyte production followed by clearance is repeated. The results obtained are not consistent with a subpopulation of individuals infected with multiple clones acting as focal infectious reservoirs for the rest of the population.

Further support for ongoing transmission through the population was the appearance of a set of new gametocyte clones on week 6 and peaking at week 8. The peak in clonal diversity coincided with a peak in gametocyte prevalence as well as parasite detection by microscopy. This could suggest the introduction of new parasite strains into the population that replicated to higher densities before being controlled, which resulted in higher numbers of infections detected by microscopy and the production of more gametocytes. Such a correlation between gametocyte prevalence and microscopic parasite prevalence has been reported previously^22,29,31,32^. However, despite the concurrent increase in microscopic parasite and gametocyte detection on week 8, overall the geometric mean gametocyte densities in both patent and subpatent infections were similar throughout the study and both populations would be expected to contribute to the infectious reservoir^5,29,33,34^. The factors regulating gametocyte production have been examined in vitro, in rodent malaria, and symptomatic infections^22,25,35–37^, but not yet carefully examined in asymptomatic infections. Even though low Hb levels have been linked to gametocyte prevalence in symptomatic infections^38^, the lack of association with Hb levels observed in this study suggest identifying differences in gametocyte production in asymptomatic infections will be an important focus for future studies.

In areas of ongoing transmission, protection against clinical disease has been associated with the development of antibodies specific for parasite specific-antigens^39–44^. Antibodies against asexual stage antigens including EBA175 RIII–V that inhibit merozoite invasion have been shown to correlate with protection^18,42,43,45^. Here IgG titers against EBA175RIII–V as well as two sexual-stage antigens, Pfs48/45.6C and Pfs230proC, were inversely correlated with parasite and gametocyte density consistent with the children having partial immunity. The specific contribution of anti-EBA175RIII–V antibodies to protection is difficult to predict from this study, especially given the significant inverse correlation between parasitemia and anti- Pfs48.45.6C and Pfs230proC IgG titers even though Pfs48/45 and Pfs230 are not expressed by asexual parasites. In fact, unless the RBCs are lysed in the human host, Pfs48.45 and Pfs230 antigens are not accessible to antibodies until the parasite emerges from the RBC as a gamete in the mosquito midgut^46–48^. Consequently, it is likely that the increased IgG titers observed are markers of a more robust general anti-parasite immune response that effectively reduces parasitemia and consequently gametocyte levels^49,50^. Further inquiry into the specific immune response associated with the clearance of different clones is needed to better understand the development of protection. At the population level, the median antibody titer against EBA175RIII–V antigen was maintained through the 10 weeks in individuals that were either persistently or sporadically infected, consistent with the longevity of these antibodies in earlier studies^46,47^. However, the 11 subjects that remained uninfected, the median EBA175RIII–V antibody levels were consistently lower at all time points.

Although this difference in anti-EBA175RIII–V antibody concentration was not significant, possibly due to the small number of persistently uninfected individuals, lower antibody levels in the uninfected volunteers could suggest that periodic exposure to antigen is needed to maintain high antibody titers. Continued follow-up through the rest of the dry season would be needed to evaluate whether levels continue to decline in the absence of reinfection. In contrast, anti- Pfs48/45.6C or Pfs230proC titers remained constant in persistently uninfected individuals as well as those that did and did not have parasites or gametocytes through the study period. This pattern is consistent with the presence of long-lived antibody-secreting plasma cells and have been observed previously^51–54^. Prior reports have suggested that persistent antigen exposure for longer than 10 weeks is needed to induce a stable long-term antibody response^24,54–56^, which could have happened during a prior malaria season. Although the immunogenicity of these antigens is well documented, immune responses have been reported to be higher in asymptomatic vs symptomatic infections, adults vs children, high vs low parasite prevalence population, and high vs low transmission intensities or zones^13,24,54,57,58^. However, the variation in antibody responses between studies makes it difficult to draw strong conclusions^59,60^, and suggest additional factors that have not yet been identified are likely to contribute to the responses. Given the persistent spread of the same set of four clones from person to person during the 10-week study period, it is unlikely that the population has strong transmission-blocking immunity. One possibility is that Pfs48.45 and Pfs230 are polymorphic in these distinct gametocyte clones^61–64^. Another reason comes from recent work screening monoclonal antibodies against Pfs48/45^65^ and Pfs230^66,67^, that suggests there are a limited number of transmission-blocking epitopes and different polyclonal serum samples contain differing levels of blocking and non-blocking antibodies. Future investigation of the infectiousness of the individuals with gametocytes and high antibody titers would continue to advance our understanding of the factors underlying transmission and guide the development of effective transmission-blocking interventions.

Together, the high prevalence of asexual and sexual parasites in school children enrolled in the study with variable carriage of distinct parasite strains at the individual level is consistent with ongoing malaria transmission in a partially immune population. The relatively low individual MOI (< 1.5) and lack of persistent gametocyte carriage by any one individual argues against a focal infectious reservoir that could be targeted for treatment. Rather, the data suggest that most of the population could contribute to transmission and is susceptible to the introduction of new parasites strains. This pattern of transmission highlights the importance of an effective vaccine to provide long-lasting immunity. However, the high prevalence of asymptomatic parasites after the peak malaria season in individuals with consistent antibody titers against asexual and sexual antigens demonstrates the challenge to vaccine development. More extensive analysis of the immune response before and after parasite clearance is needed to understand strain-specific clearance and develop strategies to induce antibodies that effectively block infection and transmission to successfully control malaria.

## Methods

### Study design, site, and population

A subset of 100 children aged between 6 to 12 years was selected from a larger cohort of an ongoing malaria transmission study. This age group was selected based on the reported high susceptibility to clinical malaria and parasitemia^68,69^. The samples used in this study were collected from November 2017 to January 2018 during the off-peak malaria transmission season. The participants were school children from two communities (50 each from Obom and Simiw) as described in our previous studies^70,71^. The communities share similar malaria transmission patterns where infections peak between June and August. The main occupation of the inhabitants from both communities is predominantly farming^4^. The sites and the population were chosen based on observed near similar infection prevalence^4^.

### Sample collection

Venous blood samples were collected fortnightly at six-time points translating into days 0, 14, 28, 42, 56, and 70. A milliliter of blood was obtained from each participant at all-time points. The samples were processed as follows; 100 μl of whole blood was preserved (Trizol) for gene transcripts analysis, 100 μl of whole blood spotted on filter paper (Whatman 3 mm) for parasite identification, about 10 μl of whole blood for Giemsa-stained smears for microscopic parasite identification and quantification. Hemoglobin levels were measured monthly (days 0, 28, and 56) by spotting 10 μl of whole blood on a Urit 12 HB meter (Accurex Biomedical, China). On-site diagnosis of *P. falciparum* infection was done by spotting 5 μl of whole blood on the HRP2 RDT kit on days 0 and 70 as well as selectively on any other sample collection day (14, 28, 42 and 56) for participants with body temperatures above 37.5 °C. The remaining whole blood was centrifuged and the plasma saved at − 20 °C for the immunological assays. The axillary body temperature of the participants was measured using a digital thermometer at each time point.

### *Plasmodium falciparum* detection and quantification by microscopy

*Plasmodium falciparum* identification and density were determined using light microscopy. The parasites were counted against 200 white blood cells (WBC) for the Giemsa stained thick smears following the standard protocols by WHO^72^, by three malaria microscopists. Due to the difficulty in detecting microscopic parasites at low parasite densities in the asymptomatic infections and to increase the sensitivity and limit of detection of the parasite in the blood films to 90% and under field conditions^73,74^, the cut-off for parasite positivity by microscopy was set at ≥ 80/µl (parasite per microliter of blood).

### Total parasite and gametocytes detection by RT-qPCR

Total parasites and gametocytes were detected by measuring *Pf18S rRNA* and *Pfs25* mRNA transcripts respectively as previously described^22,32^. Briefly, RNA was extracted from the Trizol preserved whole blood samples using the Quick RNA miniprep kit (Zymo Research, USA). DNase 1 on-column treatment was performed before RNA elution to minimize gDNA contamination. RNA was converted to cDNA using the Protoscript II first-strand cDNA synthesis kit (NEB, UK) with both oligo dT and random hexamer primers. ABI Fast SYBR Green 2X RT-PCR kit was used for the RT-PCR reactions. The amplifications were performed on ABI Quanti-Studio 5 systems (Thermo Fisher Scientific, USA). cDNA from synchronized and hemocytometer quantified NF54 (high gametocyte producer), RCM47 (gametocyte deficient) and 3D7 (low gametocytes producer) strains (matured gametocytes or early rings) served as parasite controls, while no template control (NTC) and no reverse transcriptase control (No RTC) from the cDNA prep and water were used as negative controls. Any cycle threshold (CT) value below those of the negative controls was considered positive for the specific gene. All samples were run in triplicates, and mean CT values used for the analysis were from at least duplicate readings with a standard deviation below 0.05. A standard curve using cDNA generated from RNA isolated from a known number of purified ring stage parasites or stage V gametocytes was used to calculate parasite density.

### Gametocyte diversity

Gametocyte diversity was assessed only in randomly selected samples that tested positive for *Pfs25* transcripts (gametocyte positive infections). Diversity was assessed by amplifying region 3 of the gametocyte-specific gene, *Pfg377*, from cDNA using the nested RT-PCR protocol of Menegon et al.^27^, previously described^57^. The cDNA templates were amplified using AmpliTaq Gold Fast PCR Master Mix (Applied Biosystems, USA). cDNA and gDNA from laboratory cultured gametocytes from the 3D7 *P. falciparum* parasite isolate were used as positive controls for the reactions whereas NTC, no RTC and nuclease free water were used as negative controls. Allele sizes were determined against the 50 bp (NEB, UK) ladder used on the 2% ethidium bromide-stained agarose gel.

### Immunological profiling of gametocyte and asexual antibodies

Plasma IgG and IgM of naturally induced antibody titers against *P. falciparum* recombinant Pfs48/45.6C and Pfs230proC antigens and IgG antibodies against EBA175RIII–V were measured by an indirect enzyme-linked immunosorbent assay^63,70,75^. Briefly, the antigens were diluted to 1 µg/ml and coated at 100 µl/well on a plate (Nunc MaxiSorp, UK). Recombinant polyclonal human IgG (PB055, the Binding Site) at a starting concentration of 1000 ng/ml was used as the standard calibrator to measure the total IgG. Hyper-reactive sera from individuals with high asexual [Merozoite surface protein 3 (MSP3)] or sexual (Pfs48/45) stage antibody titers were used as standard calibrators for IgM at a dilution of 1:200 (100 μl/well) and positive controls. Sera from malaria naïve individuals with low antibody titers to the test antigens were negative controls for the assays. Plasma samples and controls were diluted at 1:200 for the assays. Color development was stopped with 100 µl/well of 0.2 M H_2_SO_4_ and optical densities (OD) read at 450 nm using ELISA plate reader (Biotek, VT, USA). The measured OD values were converted into concentration using the ADAMSEL software (Edmond J. Remarque). Antibody titers (mean value from duplicate) were reported as concentrations in actual units (AU).

### Data analysis

For microscopic *P. falciparum* infections, parasite density was calculated based on the assumption of the standard 8000 leukocytes per microliter of blood from three microscopists’ readings. The cycle threshold (CT) values of the no template control were used to determine the cut-off for the presence or absence of parasite and gametocyte at submicroscopic level. The detection limit of *Pf18S* rRNA (CT ≤ 34) and *Pfs25* (CT ≤ 37) mRNA transcripts estimated 0.08 and 0.02 parasites/gametocytes per µL reference to the standard curve of parasite ring (asexual) and gametocyte stages, below the CT of the negative controls^76^. Nonlinear regression was used to assess the influence of patent infections on gametocyte positivity. The gametocyte multiplicity of infection (MOI) was estimated from the total number of gametocyte alleles in the parasite-infected population at each sampling point. Numeric values including sex, age, temperature, hemoglobin levels, *P*. *falciparum* and gametocyte prevalence were represented in counts, geometric mean (GM) or percentage (%). The difference in parasite infections, densities, gametocyte positivity and antibody (against gametocyte and asexual antigens) titers (four groups -uninfected, sporadic, persistent total infections and persistent gametocytes) at the time points were determined by non-parametric statistical methods by the Fisher’s exact Chi-squared and Kruskal Wallis tests. A p-value < 0.05 was considered statistically significant. Dynamics in the infections between the time points were analyzed using STATA (version 15). Data analyses were performed with Prism version 8.01 (GraphPad Software).

### Ethical approval

The study received ethical approval from the Institutional Review Board of the Noguchi Memorial Institute for Medical Research, University of Ghana (protocol # 024/14-15), permission from the Ghana Education Service, and the Head of the schools were also sought. Written parental informed consent was obtained from a parent or guardian before a child was enrolled. Prior to consenting and enrollment, the study’s objectives, methods, anticipated benefits, and potential hazards were explained to the parents/guardians. The parents/guardians were encouraged to ask questions and clarification about any aspect of the study that was unclear. The parents/guardians were also informed about their liberty to withdraw their children at any time point without penalty. The participant information was treated as confidential. All methods were performed in accordance with the relevant guidelines and regulations governing the study.

## Supplementary Information

Supplementary Information.
