# Supplementary material for: Persistent Plasmodium falciparum infections enhance transmission-reducing immunity development
Source: Sci Rep. 2021 Nov 1;11:21380. doi: 10.1038/s41598-021-00973-5 (PMC8560775; doi:10.1038/s41598-021-00973-5)
Supplement: Supplementary file 1 — Supplementary Information. [file 41598_2021_973_MOESM1_ESM.pdf]

Persistent *Plasmodium falciparum* infections enhance transmission-reducing immunity development

Ruth Ayanful-Torgby [1*], Esther Sarpong [2], Hamza B. Abagna [1], Dickson Donu [1], Evans Obboh [2], Benedicta A. Mensah [1], Joshua Adjah [1], Kim C. Williamson [3], Linda E. Amoah [1*]

Supplementary Table 1: Infection groups and hemoglobin levels (Day 0)

Parameter	Uninfected (n=11)	Sporadic infection (n=49)	Persistent infection (n=18)	Persistent gametocyte (n=11)	p value
Hb g/dl (Mean + SD)	12.05 ± 2.00	11.56± 1.54	11.37± 1.50	11.72±1.69	0.5793
Hb <10 (n)	0	5	2	0	

Hemoglobin levels among the uninfected, sporadically infected; ≤4 infections (n = 49), persistently infected; ≥5 infections (n = 39) and persistent gametocytes; ≥5 episodes (n=11) of gametocytemia. Infections were determined by RT-qPCR for the six sampling periods.

Supplementary Table 2: Individual infection frequency (episodes) by detection method through the course of the study

**Infection^ frequency**	**Total parasite Microscopy**	**Total parasite Molecular**	**Subpatent Infections**	**Gametocyte* Positive**
**0**	33	11	19	15
**1**	15	11	28	16
**2**	18	6	26	18
**3**	13	15	18	18
**4**	13	14	3	10
**5**	6	26	4	9
**6**	1	16	1	2

* Only *Pf 18s rRNA* positive individuals were tested for gametocytes.
^A child was excluded from the analysis due to malaria unrelated illness.

Supplementary Table 3: Gametocyte clone(s) and frequency of occurrence in the subpopulation

ID	Day 0	Day 14	Day 28	Day 42	Day 56	Day 70
S- 041	340		320	300	280, 380	200, 300
S- 137	360			340	360, 400	340
124				340	300, 340	240, 280
30				300	300	
112		320	360		300	
17				340	380	
57					180, 320	340
S- 045			340, 380		280, 360	360
S- 043	340		380, 400	280, 380		
72			340		360	340, 380
S- 005			340, 400	240, 380		
95			360		240, 300	
26		320, 340			280	320, 360
134		340	340		340, 380	
102					340, 380	300, 320
80			340		300, 360	320
7				320	300, 360	
S- 212		340, 400	340		340, 320	
S- 164		340		320		
105	340				320	
143	340				300	
146		340				340
10	320				300	
89		300			340	

Gametocyte clones in 24 individual that had *Pfg377* genotypes assessed at two or more time points
ID S-041 to S-212. Gametocyte clones in 18 individual that had *Pfg377* clones in 2 consecutive samples.
ID S-164 to 89. Gametocyte clones in 6 individual that had *Pfg377* clones at two or more time points.
The red numbers indicate the same *Pfg377* clone was detected at 2 consecutive time points.

Color	Key
	Pfg377 not assessed
	No gametocyte
	Not infected
	No sample collected

Supplementary Table 4: P values for the comparison of the anti-gametocyte and asexual antigen titers in the four infection groups (Uninfected, Sporadic infections, Persistent infections, and Persistent gametocytes) * at the indicated time point.

Time point	IgG Pfs48/45	IgM Pfs48/45	IgG Pfs230	IgM Pfs230	IgG EBA175
D14	0.4592	0.1908	0.4287	0.9939	0.0631
D42	0.2728	0.6723	0.4228	0.6018	0.1276
D70	0.2742	0.0931	0.9610	0.2510	0.1134
^All time points	**0.2025**	**0.4416**	**0.1647**	**0.7274**	**0.3651**

*Kruskal-Wallis (one-way ANOVA) was used to determine the p values for differences in titer among the four infection groups shown in Figs 3 & 4 (uninfected, sporadically infected, ≤4 infections (n = 49), persistently infected, ≥5 infections (n = 39) and persistent gametocytes, ≥5 episodes (n=11) of gametocytemia).

^p values in bold represent the population antibody titers on the three time points (Days 14, 42 and 70).

Supplementary Table 5: P-values for the comparison of sexual and asexual antibody titers among infections with gametocytes, without gametocytes and the uninfected individuals at the indicated time points.

Time point	IgG Pfs48/45	IgM Pfs48/45	IgG Pfs230	IgM Pfs230	IgG EBA175
D14	0.8399	0.7506	0.1618	0.7675	0.8118
D42	0.6524	0.9460	0.1719	0.7875	0.7214
D70	0.4472	0.2046	0.5066	0.0538	0.062

Kruskal-Wallis (one-way ANOVA) was used to determine the p values for differences in titer among there groups (infected with gametocytes, infected without gametocytes and uninfected individuals).

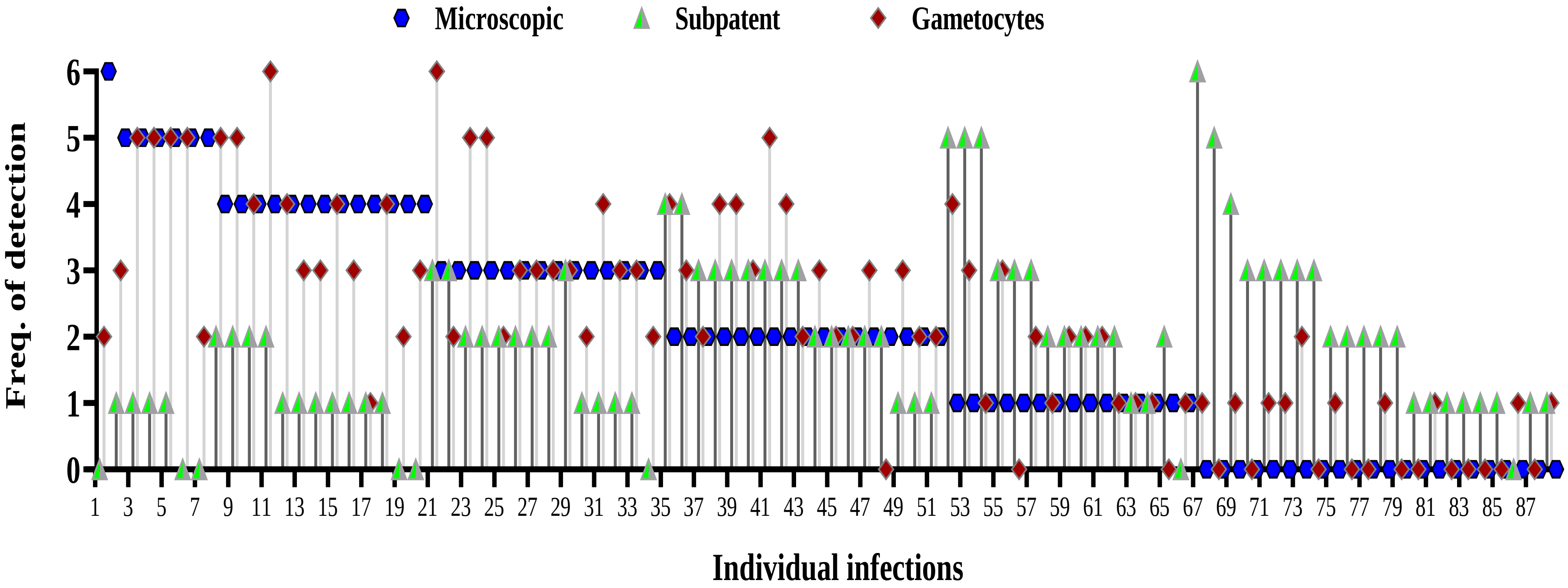

Supplementary Fig 1: Infection dynamics and frequency at individual level

The frequency of detection of parasites using microscopy (Micro, blue hexagon), subpatent infections using *Pf 18srRNA* RT-qPCR (Subpatent, green and gray arrowhead), or gametocytes using *Pfs25* RT-qPCR (Gametocyte, red diamond) in each individual. Individuals are arranged by decreasing order of microscopic detection frequency. The eleven individuals who were never infected were excluded.

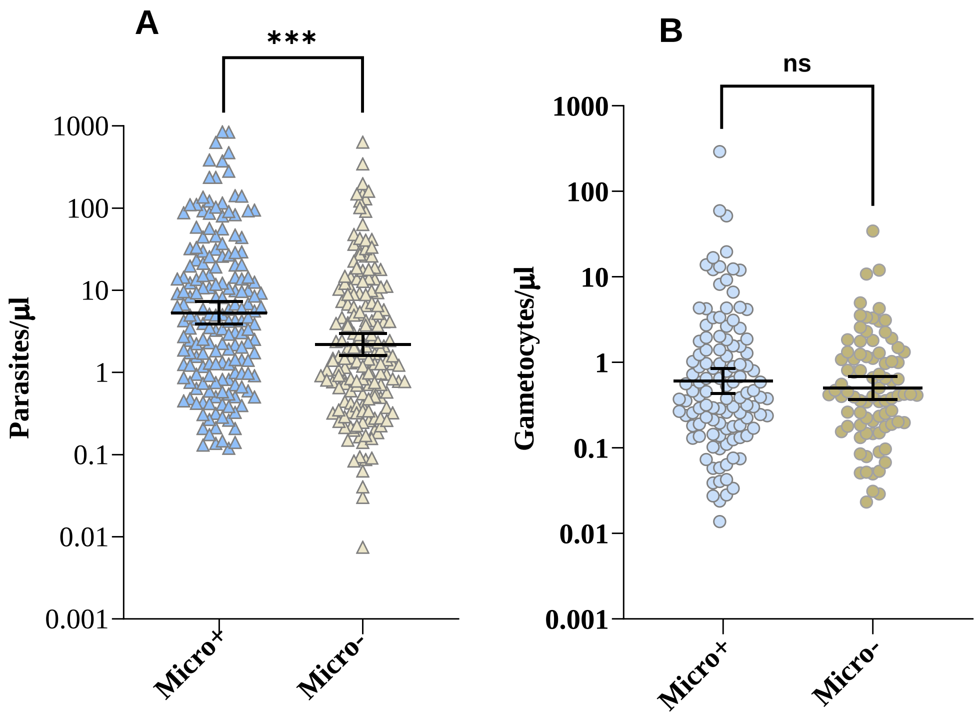

Supplementary Fig. 2: Total parasite and gametocyte quantification by RT-qPCR in samples

Parasite (*Pf18S rRNA*, RT-qPCR) and gametocyte (*Pfs25*, RT-qPCR) densities in samples with (Micro+) or without (Micro-) patent/microscopic parasites. The estimated total parasite densities in all infected samples (**A)** and gametocyte densities (**B**) in 1 ul of blood sample were calculated using a standard curve. Mann Whitney test t-test was used to compare densities in the samples expressed in geometric mean at 95% confidence limit (CI). ns $p > 0.05$, ***$p \leq 0.001$.

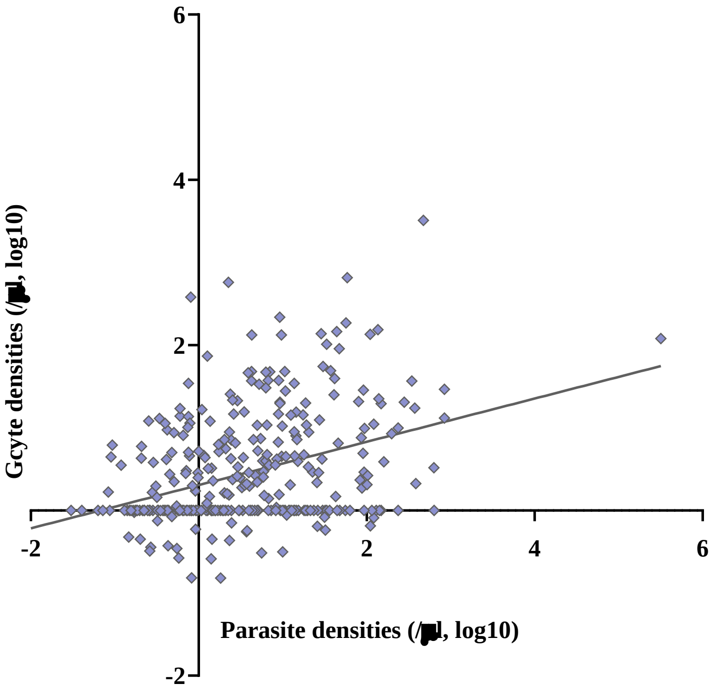

Supplementary Fig. 3: Gametocyte densities correlated positively with the parasite in the samples.

Comparison of gametocyte and parasite densities estimated from *Pfs25* and *Pf18s rRNA* RT-qPCR analysis. A positive correlation between parasite and gametocyte densities was observed (Pearson r= 0.3691, p ≤ 0.0001).
